# Secondary range symmetric matrices

**DOI:** 10.12688/f1000research.144171.1

**Published:** 2024-02-19

**Authors:** Divya Shenoy

**Affiliations:** 1 Department of Mathematics, Manipal Institute of Technology, Manipal, Manipal Academy of Higher Education, Udupi, Karnataka, 576104, India

**Keywords:** Generalized inverses, Secondary generalized inverses, Secondary transpose, EP matrices

## Abstract

The concept of secondary range symmetric matrices are introduced here. Some characterizations as well as the equivalent conditions for a range symmetric matrix to be secondary range symmetric matrix is given. The idea of range symmetric matrices, range symmetric matrices over Minkowski space and secondary range symmetric matrices are different, and is depicted with the help of suitable examples. Finally, a necessary and sufficient condition for a secondary range symmetric matrix to have a secondary generalized inverse has been obtained.

## Introduction

The theory of symmetric matrices as well as range symmetric matrices are well known in literature. A matrix is said to be EP (or range symmetric), whenver the range space of the matrix is equal to the range space of its conjugate transpose. In other words, matrix is EP whenever its null space is same as that of the null space of its conjugate transpose. Ballantine ^1^ has studied about the product of two EP matrices of specific rank to be again an EP matrix. In ^2^ new characterizations of EP matrices are given. Also, weighted EP matrix is defined and characterized. Meenakshi ^3^ extended the concept of range symmetric matrices over Minkowski space. In 2014, the same author defined range symmetric matrices in indefinite inner product space. ^4^

For an n×n matrix A , the secondary transpose is related to transpose of the matrix by the relation $A^{S}={\mathrm{VA}}^{T}V$ . Here, the matrix V has non zero unitary entries only on the secondary diagonal. For a matrix A with complex entries, the secondary transpose will be renamed as secondary conjugate transpose $A^{\theta }$ and is given by $A^{\theta }={\mathrm{VA}}^{*}V$ .

Definition 1
(Ref. 5) *Let* $A\in {\mathbb{R}}^{n\times n}$ *. Then the secondary transpose (or secondary conjugate transpose* $A^{\theta }$ *in the case of complex matrices) of* A *denoted by* $A^{S}$ *and is defined as* $A^{S}=\left(b_{\mathrm{ij}}\right)$ *, where* $b_{\mathrm{ij}}=a_{n-j+1,n-i+1}$ *,* i,j=1,2,…n *.*

Throughout this article we assume V to be a permutation matrix with units in the secondary diagonal. Also, $N\left(A\right)$ represents the null space of the matrix A . The column space and rank of A are denoted by $C\left(A\right),\rho \left(A\right)$ respectively. The set of matrices of order n×n over the field of real numbers is denoted by ${\mathbb{R}}^{n\times n}$ .

The concept of secondary conjugate transpose is gaining importance in recent years. Shenoy ^6^ has defined Outer Theta inverse by combining the outer inverse and secondary transpose of a matrix A . Drazin-Theta matix $A^{D,\theta }$ ^7^ is a new class of generalized inverse introduced for a square matrix of index m. One can refer ^8^ for the extension of these inverses over rectangular matrices. R. Vijayakumar ^9^ introduced the concept of secondary generalized inverse with the help of secondary transpose of a matrix. This concept is similar to Moore Penrose inverse. But unlike Moore penrose inverse, existence of s-g inverse is not assured in general. In Ref. 10, a necessary and sufficient conditions of existence of s-g invese is given. In the same article, a few characterizations and a determinantal formula for s-g inverse also has been discussed. In 2009, Krishnamoorthy and Vijayakumar ^11^ has defined the concept of s-normal matrices with the help of secondary transopse for a class of complex square matrices. Jayashree ^12^ has defined secondary k-range symmetric fuzzy matrices. Its relation with s-range symmetric fuzzy matrices, k-range fuzzy symmetric matrices and EP matrices are defined.

In this article, we define secondary range symmetric matrices. Several equivalent conditions for a matrix to be secondary range symmetric, is obtained here. Also, the existence of secondary generalized inverse of a secondary range symmetric matrix is discussed.

Below are some useful deifnitions and results related to secondary conjugate transpose.

Definition 2
(Ref. 13) *Let* $A\in {\mathbb{C}}^{n\times n}$ *. Then the conjugate secondary transpose of* A *denoted by* $A^{\theta }$ *and is defined as* $A^{\theta }={\bar{A}}^{s}=\left(c_{\mathrm{ij}}\right)$ *where* $c_{\mathrm{ij}}={\bar{a}}_{n-j+1,n-i+1}$ *.*

Definition 3
(Ref. 13) *A matrix is said to be secondary normal (s-normal) if* ${\mathrm{AA}}^{S}=A^{S}A$ *.*

Definition 4
(Ref. 10) $A^{{†}_{S}}$ *is said to be secondary generalized inverse of* A *if*

$${\mathrm{AA}}^{{†}_{S}}A=A\;A^{{†}_{S}}{\mathrm{AA}}^{{†}_{S}}=A^{{†}_{S}}$$

*and* ${\mathrm{AA}}^{{†}_{S}}$ *and* $A^{{†}_{S}}A$ *are* S - *symmetric.*

Theorem 0.1
(Ref. 10) *Given an* m×n *matrix* A *. The following statements are equivalent.*

(i)
A *has s-cancellation property (i.e.,* $A^{s}\mathrm{AX}=0\mathrm{AX}=0$ *and* ${\mathrm{YAA}}^{s}=0\mathrm{YA}=0$ *).*
(ii)
$\rho \left(A^{s}A\right)=\rho \left({\mathrm{AA}}^{s}\right)=\rho \left(A\right)$

(iii)
$A^{s}$ *exists.*

Definition 5
(Ref. 14) *A matrix* $A\in {\mathbb{C}}^{n\times n}$ *is said to be EP (or range symmetric) if* $N\left(A\right)=N\left(A^{*}\right)$ *.*

Meenakshi ^3^ has defined EP in Minkowski space and has given equivalent conditions for a matrix to be range symmetric.

Definition 6
(Ref. 3) *A matrix* $A\in {\mathbb{C}}^{n\times n}$ *is said to be range symmetric in Minkowski space if and only if* $N\left(A\right)=N\left(A^{+}\right)$ *.*

Here $A^{+}$ represents the Minkowski adjoint given by $A^{+}={\mathrm{GA}}^{*}G$ where G is the Minkowski metric tensor.

## Results

In this section, we define secondary range symmetric matrices which is analogous to that of range symmetric matrices. Some equivalent conditions for a matrix to be range symmetric is also given here.

Definition 7
X *is secondary right (left) normalized g inverse of* A *if* AXA=A *,* XAX=X *and* AX *is* S *-symmetric. (* XA *is* S *-symmetric).*

Definition 8
*Consider* $A\in {\mathbb{R}}^{m\times n}$ *. The s-transpose of* A *is defined as* $A^{s}=\left(b_{\mathrm{ij}}\right)$ *where* $b_{\mathrm{ij}}=a_{m-j+1,n-i+1}$ *,* 1≤i≤n,1≤j≤m *.*

Definition 9
*A matrix* $A\in {\mathbb{R}}^{n\times n}$ *secondary range symmetric if and only if* $N\left(A\right)=N\left(A^{S}\right)$

Theorem 0.2
*Let* $A\in {\mathbb{R}}^{n\times n}$ *. Then the following conditions are equivalent.*

(i)
A *is secondary range symmetric*
(ii)
VA *is EP*
(iii)
AV *is EP*
(iv)
$N\left(A^{*}\right)=N\left(\mathrm{AV}\right)$

(v)
$C\left(A\right)=C\left(A^{S}\right)$

(vi)
$A^{S}=\mathrm{BA}=\mathrm{AC}$ *where* B *and* C *are some nonsingular matrices*
(vii)
$C\left(A^{*}\right)=C\left(\mathrm{VA}\right)$

(viii)
$C\left(A^{*}\right)⊕N\left(A\right)={\mathbb{C}}^{n}$

(ix)
$C\left(A\right)⊕N\left(A^{*}\right)={\mathbb{C}}^{n}$

Proof 1
$$\begin{array}{l} \text{A is secondary range symmetric}\Leftrightarrow N\left(A\right)=N\left(A^{S}\right)\\ \;\Leftrightarrow N\left(\mathrm{VA}\right)=N\left(A^{S}V\right)\\ \;\Leftrightarrow \text{VA is EP}\\ \;\Leftrightarrow V\left(\mathrm{VA}\right)V^{S}\text{is EP}\\ \;\Leftrightarrow \text{AV is EP}. \end{array}$$

*Hence* $\left(1\right)\Leftrightarrow \left(2\right)\Leftrightarrow \left(3\right)$ *holds true.*

$\left(1\right)\Leftrightarrow \left(4\right)$

$$\begin{array}{l} \text{A is secondary range symmetric}\Leftrightarrow N\left(A\right)=N\left(A^{S}\right)\\ \;\Leftrightarrow N\left(A\right)=N\left({\mathrm{VA}}^{*}V\right)\\ \;\Leftrightarrow N\left(A\right)=N\left(A^{*}V\right)\\ \;\Leftrightarrow A^{*}V=A^{*}{\mathrm{VA}}^{\left(1\right)}A\\ \;\Leftrightarrow A^{*}=A^{*}{\mathrm{VA}}^{\left(1\right)}\mathrm{AV}\\ \;\Leftrightarrow A^{*}=A^{*}{\left(\mathrm{AV}\right)}^{\left(1\right)}\mathrm{AV}\\ \;\Leftrightarrow N\left(A^{*}\right)=N\left(\mathrm{AV}\right) \end{array}$$

*This proves the equivalence of* $\left(1\right)$ *and* $\left(4\right)$ *.*

$\left(3\right)\Leftrightarrow \left(5\right)$

$$\begin{array}{l} \text{AV is range symmetric}\Leftrightarrow C\left(\mathrm{AV}\right)=C{\left(\mathrm{AV}\right)}^{*}\\ \;\Leftrightarrow C\left(A\right)=C\left({\mathrm{VA}}^{*}\right)\\ \;\Leftrightarrow C\left(A\right)=C\left({\mathrm{VA}}^{*}V\right)\\ \;\Leftrightarrow C\left(A\right)=C\left(A^{S}\right) \end{array}$$

*Hence* $\left(3\right)$ *and* $\left(5\right)$ *are equivalent.*

$\left(2\right)\Leftrightarrow \left(6\right)$

$$\begin{array}{l} \mathrm{VA}\;\text{is range symmetric}\Leftrightarrow {\left(\mathrm{VA}\right)}^{*}=\mathrm{VAP}\;\text{for some nonsingular}\;n\times n\;\text{matrix}\;P\\ \;\Leftrightarrow A^{*}V=\mathrm{VAP}\\ \;\Leftrightarrow {\mathrm{VA}}^{*}V=\mathrm{AP}\\ \;\Leftrightarrow A^{S}=\mathrm{AP}\\ \;\Leftrightarrow A={\left(\mathrm{AP}\right)}^{S}=P^{S}A^{S}\\ \;\Leftrightarrow A^{S}={\left(P^{S}\right)}^{-1}A\\ \;\Leftrightarrow A^{S}=\mathrm{KA}\;\text{where}\;K={\left(P^{S}\right)}^{-1}. \end{array}$$

*Hence* $\left(2\right)\Leftrightarrow \left(6\right)$ .

$\left(5\right)\Leftrightarrow \left(7\right)$

$$\begin{array}{l} C\left(A\right)=C\left(A^{S}\right)\Leftrightarrow C\left(A\right)=C\left({\mathrm{VA}}^{S}V\right)\\ \;\Leftrightarrow C\left(A\right)=C\left({\mathrm{VA}}^{S}\right)\\ \;\Leftrightarrow {\mathrm{VA}}^{*}={\mathrm{AA}}^{\left(1\right)}{\mathrm{VA}}^{*}\\ \;\Leftrightarrow A^{*}={\mathrm{VAA}}^{\left(1\right)}{\mathrm{VA}}^{*}\\ \;\Leftrightarrow A^{*}=\left(\mathrm{VA}\right){\left(\mathrm{VA}\right)}^{\left(1\right)}A^{*}\\ \;\Leftrightarrow C\left(A^{*}\right)=C\left(\mathrm{VA}\right) \end{array}$$

*Thus equivalence of* $\left(5\right)$ *and* $\left(7\right)$ *are proved.*

$\left(2\right)$ ⇔ $\left(8\right)$ .

$$\begin{array}{l} \text{VA is range symmetric}\Leftrightarrow {\mathbb{C}}^{n}=C\left(\mathrm{VA}\right)⊕N\left(\mathrm{GA}\right)\\ \;=C\left({\left(\mathrm{VA}\right)}^{*}\right)⊕N\left(A\right)\\ \;=C\left(A^{*}V\right)⊕N\left(A\right)\\ \;=C\left(A^{*}\right)⊕N\left(A\right) \end{array}$$

*Thus equivalence of* $\left(2\right)$ *and* $\left(8\right)$ *are proved.*

$\left(3\right)\Leftrightarrow \left(9\right)$

$$\begin{array}{l} \text{AV is range symmetric}\Leftrightarrow {\mathbb{C}}^{n}=C\left(\mathrm{AV}\right)⊕N\left(\mathrm{AV}\right)\\ \;={\mathbb{C}}^{n}=C\left(\mathrm{AV}\right)⊕N\left({\left(\mathrm{AV}\right)}^{*}\right)\\ \;=C\left(A\right)⊕N\left(A^{*}\right) \end{array}$$

*Thus the equivalence of* $\left(3\right)$ *and* $\left(9\right)$ *is proved.*

From the following example, it is clear that EP matrices in Minkowski space defined by Meenakshi ^3^ and secondary range symmetric matrices are two different concepts.

Consider a matrix $A=\left(\begin{array}{cc} -1 & 1\\ 1 & -1 \end{array}\right)$ where $A^{S}=\left(\begin{array}{cc} -1 & 1\\ 1 & -1 \end{array}\right)$ . Here, A is secondary normal as well as $\rho \left(A\right)=\rho \left({\mathrm{AA}}^{S}\right)$ . The matrix A is secondary range symmetric. However, the matrix is not range symmetric in Minkowski’s space since $A^{+}={\mathrm{GA}}^{*}G=\left(\begin{array}{cc} -1 & -1\\ -1 & -1 \end{array}\right)$ . It is clear that $N\left(A\right)\neq N\left(A^{+}\right)$ .

Let $B=\left(\begin{array}{cc} 1 & -1\\ 1 & -1 \end{array}\right)$ and $B^{S}=\left(\begin{array}{cc} -1 & -1\\ 1 & 1 \end{array}\right)$ . Clearly B is not secondary range symmetric.

Observe that B is range symmetric in Minkowski space. Since, $B^{+}={\mathrm{GB}}^{*}G=\left(\begin{array}{cc} 1 & -1\\ 1 & -1 \end{array}\right)$ so that $N\left(B\right)=N\left(B^{+}\right)$ .

Note that $A^{S}={\mathrm{VA}}^{*}V={\mathrm{VGA}}^{+}\mathrm{GV}$ .

A necessary condition for a matrix to be a s-EP (secondary range symmetric) is proved here.

Theorem 0.3
*Let* $A\in {\mathbb{R}}^{n\times n}$ *. If* A *is secondary normal and* $\rho \left(A\right)=\rho \left({\mathrm{AA}}^{S}\right)$ *, then* A *is secondary range symmetric.*

Proof 2
*Since* A *is secondary normal,* ${\mathrm{AA}}^{S}=A^{S}A$ *. Hence* $\rho \left(A\right)=\rho \left({\mathrm{AA}}^{S}\right)=\rho \left(A^{S}A\right)=\rho \left(A^{S}\right)$ *which implies* $N\left(A\right)=N\left({\mathrm{AA}}^{S}\right)=N\left(A^{S}A\right)=N\left(A^{S}\right)$ *. Thus* A *is secondary range symmetric.*

A relation connecting range symmetric and secondary range symmetric matrices is given below:

Theorem 0.4
*Let* $A\in {\mathbb{R}}^{n\times n}$ *. Then any two of the following conditions imply the third one.*

(1)
A *is EP.*
(2)
A *is secondary EP.*
(3)
$C\left(A\right)=C\left(\mathrm{VA}\right)$ *.*

Proof 3
$\left(1\right),\left(2\right)\Rightarrow \left(3\right)$

*Since* A *is EP,* $C\left(A\right)=C\left(A^{*}\right)$ *. By* *Theorem 0.2* *,* $C\left(A^{*}\right)=C\left(\mathrm{VA}\right)$ *as* A *is secondary range symmetric. Hence* A *is secondary EP* ⇔ $C\left(A\right)=C\left(\mathrm{VA}\right)$ *.*

$\left(2\right),\left(3\right)\Rightarrow \left(1\right)$

*Since* A *is s-EP, by* *Theorem 0.2* *,* VA *is range symmetric. Hence* $C\left(\mathrm{VA}\right)=C\left({\left(\mathrm{VA}\right)}^{*}\right)=C\left(A^{*}V\right)=C\left(A^{*}\right)$ *. Also, By (3),* $C\left(A\right)=C\left(\mathrm{VA}\right)$ *from which it follows that* $C\left(A\right)=C\left(A^{*}\right)$ *. Hence* A *is range symmetric. Thus (1) holds.*

For any square complex matrix A , there exists unique S - symmetric matrices such that A=M+iN where $M=\frac{1}{2}\left(A+A^{S}\right)$ and $N=\frac{1}{2i}\left(A-A^{S}\right)$ . In the following theorem, an equivalent condition for a matrix A to be secondary range symmetric is obtained interms of M , the S -symmetric part of A .

Theorem 0.5
*For* $A\in {\mathbb{R}}^{n\times n}$ *,* A *is secondary range symmetric if and only if* $N\left(A\right)\subseteq N\left(M\right)$ *where* M *is the* S *-symmetric part of* A *.*

Proof 4
*If* A *is secondary range symmetric, then* $N\left(A\right)=N\left(A^{S}\right)$ *. For* $x\in N\left(A\right)$ *,* Ax=0 *and* $A^{S}x=0$ *. Hence* Mx=0 *. Thus,* $N\left(A\right)\subseteq N\left(M\right)$ *, then* Ax=0⇒Mx=0 *and hence* Nx=0 *. Therefore* $N\left(A\right)\subseteq N\left(N\right)$ *. Thus* $N\left(A\right)\subseteq N\left(M\right)\cap N\left(N\right)$ *. Since, both* M *and* N *are* S *-symmetric, they are secondary range symmetric.*

$$N\left(M\right)=N\left(M^{S}\right)=N\left({\mathrm{VM}}^{*}V\right)=N\left(M^{*}V\right)$$

*and*

$$N\left(N\right)=N\left(N^{S}\right)=N\left({\mathrm{VN}}^{*}V\right)=N\left(N^{*}V\right)$$

*Now*, $N\left(A\right)\subseteq N\left(M\right)\cap N\left(N\right)=N\left(M^{*}V\right)\cap N\left(N^{*}V\right)\subseteq N\left[\left(M^{*}+{\text{iN}}^{*}\right)V\right]=N\left(A^{*}V\right)$ and $\rho \left(A\right)=\rho \left(A^{*}\right)=\rho \left(A^{*}V\right)$ . *Therefore*, $N\left(A\right)=N\left(A^{*}V\right)=N\left({\mathrm{VA}}^{*}V\right)=N\left(A^{S}\right)$ . *Thus* A *is secondary range symmetric.*

We shall discuss the existence of secondary generalized inverse inverse of a secondary range symmetric matrix. First, we shall prove certain lemmas, to simplify the proof of the main result.

Lemma 1
*For an* m×n *matrix* A *, if* $A^{{†}_{S}}$ *exists, then* $C\left(A^{{†}_{S}}\right)=C\left(A^{S}\right)$ *.*

Proof 5
*If* $A^{{†}_{S}}$ *exists, then* $A^{{†}_{S}}A={\left(A^{{†}_{S}}A\right)}^{S}=A^{S}{\left({\mathrm{AA}}^{{†}_{S}}\right)}^{S}{\left(A^{{†}_{S}}\right)}^{S}$ *and*

$$A^{{†}_{S}}=A^{{†}_{S}}{\mathrm{AA}}^{{†}_{S}}=A^{S}{\left(A^{{†}_{S}}\right)}^{S}A^{{†}_{S}}\Rightarrow C\left(A^{{†}_{S}}\right)\subseteq C\left(A^{S}\right).$$

*Further*, $\rho \left(A^{{†}_{S}}\right)=\rho \left(A\right)=\rho \left(A^{S}\right)$ . *Thus*, $C\left(A^{{†}_{S}}\right)=C\left(A^{S}\right)$ .

Lemma 2
*For an* m×n *matrix* A *, if* $A^{{†}_{S}}$ *exists, then* $C\left({\mathrm{AA}}^{{†}_{S}}\right)$ *is the projection on* $C\left(A^{{†}_{S}}\right)$ *and* $A^{{†}_{S}}A$ *is the projection on* $C\left(A^{{†}_{S}}\right)$ *.*

Proof 6
$x\in C\left(A\right)$ *if and only if* $x=\mathrm{Ay}={\mathrm{AA}}^{{†}_{S}}\mathrm{Ay}={\mathrm{AA}}^{{†}_{S}}x$ *. By* *definition 2* *,* ${\mathrm{AA}}^{{†}_{S}}$ *being* S *-symmetric, idempotent is the projection on* $C\left(A\right)$ *. Similarly,* $x\in C\left(A^{{†}_{S}}\right)$ *if and only if* $x=A^{{†}_{S}}{\mathrm{AA}}^{{†}_{S}}y=A^{{†}_{S}}\mathrm{Ax}$ *and* $A^{{†}_{S}}A$ *is* S *-symmetric and idempotent. Hence* $A^{{†}_{S}}A$ *is the projection on* $C\left(A^{{†}_{S}}\right)$ *.*

Theorem 0.6
*For an* n×n *matrix* A *, the following are equivalent:*

(1)
A *is secondary range symmetric and* $\rho \left(A\right)=\rho \left(A^{2}\right).$

(2)
$A^{{†}_{S}}$ *exists and* $A^{{†}_{S}}$ *is secondary range symmetric.*
(3) *There exists a symmetric idempotent matrix E such that* AE=EA *and* $C\left(A\right)=C\left(E\right).$

Proof 7
$\left(1\right)\Rightarrow \left(2\right)$ *. Since* $\rho \left(A\right)=\rho \left(A^{2}\right)$ *and* A *is secondary symmetric, by using* *Theorem 0.2* *we have,* $\rho \left(A^{S}A\right)=\rho \left({\mathrm{BA}}^{2}\right)=\rho \left(A^{2}\right)=\rho \left(A\right)$ *and* $\rho \left({\mathrm{AA}}^{S}\right)=\rho \left(A^{2}C\right)=\rho \left(A^{2}\right)=\rho \left(A\right)$ *. Thus* $\rho \left(A\right)=\rho \left({\mathrm{AA}}^{S}\right)=\rho \left(A^{S}A\right)$ *. Hence by* *Thoerem 0.1* *, it follows that* $A^{{†}_{S}}$ *exists, By* *Lemma 1* *and* *Theorem 0.2* *,* $C\left(A^{{†}_{S}}\right)=C\left(A^{S}\right)=C\left(A\right)=C\left({\left(A^{S}\right)}^{S}\right)=C\left({\left(A^{{†}_{S}}\right)}^{S}\right)$ *. Hence* $A^{{†}_{S}}$ *is range symmetric. Thus (2) holds.*

$\left(2\right)\Rightarrow \left(3\right)$ . *Since* $A^{{†}_{S}}$ *exists, by* Lemma 1, $C\left(A^{{†}_{S}}\right)=C\left(A^{S}\right)$ , by Theorem 0.2, $A^{{†}_{S}}$ *is secondary range symmetric which implies that* $C\left(A^{{†}_{S}}\right)=C\left({\left(A^{{†}_{S}}\right)}^{S}\right)$ . *Hence* $C\left(A^{S}\right)=C\left({\left(A^{{†}_{S}}\right)}^{S}\right)$ . By Lemma 2, *it follows that* $A^{S}{\left(A^{S}\right)}^{{†}_{S}}={\left(A^{{†}_{S}}\right)}^{S}A^{S}$ , *hence* ${\left(A^{{†}_{S}}A\right)}^{S}={\left({\mathrm{AA}}^{{†}_{S}}\right)}^{S}$ . *By* definition 2, $A^{{†}_{S}}A={\mathrm{AA}}^{{†}_{S}}=E$ , *is* S - *symmetric, idempotent and* AE=EA=A ; *hence* $C\left(A\right)\subseteq C\left(E\right)$ and $\rho \left(E\right)=\rho \left({\mathrm{AA}}^{{†}_{S}}\right)=\rho \left(A\right)$ , *which implies* $C\left(A\right)=C\left(E\right)$ . *Thus* $\left(3\right)$ *holds.*

$\left(3\right)\Rightarrow \left(1\right)$ . *Since*, E is S - *symmetric and idempotent*, $E^{S}=E=E^{2}$ , *by* lemma 1.1, $E^{{†}_{S}}$ *exists and* $E^{{†}_{S}}=E$ *implies* E *is the projection on* $C\left(A\right)$ . *For all reflexive g-inverses* $A^{r}$ of A , ${\mathrm{AA}}^{r}={\mathrm{EE}}^{{†}_{S}}=E$ . *Since* E *is* S - *symmetric and idempotent*, ${\mathrm{AA}}^{r}$ *is* S - *symmetric. Hence by* definition 7, $A^{n}$ *exists and* ${\mathrm{AA}}^{n}={\mathrm{EE}}^{{†}_{S}}=E$ *which implies* EA=A . *By hypothesis* AE=EA=A . *Therefore* ${\mathrm{AA}}^{n}=A^{n}A=E$ . *Thus both* ${\mathrm{AA}}^{n}$ *and* $A^{n}A$ are S - *symmetric.* *By* definition 2, $A^{{†}_{S}}$ *exists and* $E={\mathrm{AA}}^{{†}_{S}}=A^{{†}_{S}}A$ . *By taking secondary transpose on* AE=EA=A , *we* get ${\mathrm{EA}}^{S}=A^{S}E=A^{S}$ . $C\left(A^{S}\right)\subseteq C\left(E\right)=C\left(A\right)$ *and* $\rho \left(A^{S}\right)=\rho \left(A\right)$ . *Therefore* $C\left(A\right)=C\left(A^{S}\right)$ . *By* theorem 0.2, A *is secondary ramge symmetric.* $\rho \left({\mathrm{AA}}^{S}\right)\geq \rho \left({\mathrm{AA}}^{S}{\left(A^{{†}_{S}}\right)}^{S}\right)=\rho \left(A{\left(A^{{†}_{S}}\right)}^{S}\right)$ , $\rho \left(\mathrm{AE}\right)=\rho \left(A\right)\geq \rho \left({\mathrm{AA}}^{S}\right)$ . *Thus* $\rho \left(A\right)=\rho \left({\mathrm{AA}}^{S}\right)=\rho \left(A^{2}C\right)=\rho \left(A^{2}\right)$ . *Thus* (1) *holds.* *Hence the theorem.*

Corollary 1
*Consider an* n×n *secondary range symmetric matrix* A *. Then* $A^{{†}_{S}}$ *exists if and only if* $\rho \left(A\right)=\rho \left(A^{2}\right)$ *.*

Proof 8
*Since* A *is secondary range symmetric and* $\rho \left(A\right)=\rho \left(A^{2}\right)$ *, the existence of* $A^{{†}_{S}}$ *follows from Thereom 0.6. Conversly, if* A *is secondary range symmetric, and* $A^{{†}_{S}}$ *exists, then by Theorem 0.1* $\rho \left(A\right)=\rho \left({\mathrm{AA}}^{S}\right)=\rho \left(A^{S}A\right)$ *and by* *Theorem 0.2* *,* $A^{S}=\mathrm{AC}$ *. Hence* $\rho \left(A\right)=\rho \left(\mathrm{AAC}\right)=\rho \left(A^{2}\right)$ *.*

## Conclusion

As an extension of this work, one can think of defining weighted secondary EP matrices and its characteriations. Also, extending secodary range symmetric matrix to indefinite inner prodcut spaces will open up a new area of research.
