# Secondary range symmetric matrices

**DOI:** 10.12688/f1000research.144171.2

**Published:** 2024-09-11

**Authors:** Divya Shenoy

**Affiliations:** 1 Department of Mathematics, Manipal Institute of Technology, Manipal, Manipal Academy of Higher Education, Udupi, Karnataka, 576104, India

**Keywords:** Generalized inverses, Secondary generalized inverses, Secondary transpose, EP matrices

## Abstract

The concept of secondary range symmetric matrices is introduced here. Some characterizations as well as the equivalent conditions for a range symmetric matrix to be secondary range symmetric matrix is given. The idea of range symmetric matrices, range symmetric matrices over Minkowski space and secondary range symmetric matrices are different, and is depicted with the help of suitable examples. Finally, a necessary and sufficient condition for a secondary range symmetric matrix to have a secondary generalized inverse has been obtained.

## Introduction

The theory of symmetric matrices as well as range symmetric matrices are well known in literature. A matrix is said to be EP (or range symmetric), whenever the range space of the matrix is equal to the range space of its conjugate transpose. In other words, matrix is EP whenever its null space is same as that of the null space of its conjugate transpose. Ballantine ^1^ has studied about the product of two EP matrices of specific rank to be again an EP matrix. In ^2^ new characterizations of EP matrices are given. Also, weighted EP matrix is defined and characterized. Meenakshi ^3^ extended the concept of range symmetric matrices over Minkowski space. In 2014, the same author defined range symmetric matrices in indefinite inner product space. ^4^ In Ref. 5, the concept of EP matrices to bounded operator with closed range is defined on a Hilbert space. For more characterizations of EP and hypo EP operators one can refer. ^6^^,^^7^

For an n×n matrix A , the secondary transpose is related to transpose of the matrix by the relation $A^{S}={\mathrm{VA}}^{T}V$ . Here, the matrix V has non zero unitary entries only on the secondary diagonal. For a matrix A with complex entries, the secondary transpose will be renamed as secondary conjugate transpose $A^{\theta }$ and is given by $A^{\theta }={\mathrm{VA}}^{*}V$ .

The concept of secondary conjugate transpose is gaining importance in recent years. Shenoy ^8^ has defined Outer Theta inverse by combining the outer inverse and secondary transpose of a matrix A . Drazin-Theta matix $A^{D,\theta }$ ^9^ is a new class of generalized inverse introduced for a square matrix of index m. One can refer ^10^ for the extension of these inverses over rectangular matrices. R. Vijayakumar ^11^ introduced the concept of secondary generalized inverse with the help of secondary transpose of a matrix. This concept is similar to Moore Penrose inverse. But unlike Moore penrose inverse, existence of s-g inverse is not assured in general. In Ref. 12, a necessary and sufficient conditions of existence of s-g invese is given. In the same article, a few characterizations and a determinantal formula for s-g inverse also has been discussed. In 2009, Krishnamoorthy and Vijayakumar ^13^ has defined the concept of S - normal matrices with the help of secondary transopse for a class of complex square matrices. Jayashree ^14^ has defined secondary k - range symmetric fuzzy matrices. Its relation with S - range symmetric fuzzy matrices, k - range fuzzy symmetric matrices and EP matrices are defined.

In this article, we define secondary range symmetric matrices. Several equivalent conditions for a matrix to be secondary range symmetric, is obtained here. Also, the existence of secondary generalized inverse of a secondary range symmetric matrix is discussed.

Below are some useful deifnitions and results related to secondary conjugate transpose.

## Preliminaries

The set of all n×n matrices with complex entries are denoted by $C^{n\times n}$ ( $R^{n\times n}$ for matrices with real entries. Also, $N\left(A\right)$ represents the null space of the matrix *A*. The column space and rank of *A* are denoted by $C\left(A\right),\rho \left(A\right)$ respectively.

Definition 1
(Ref. 15) *Let* $A\in {\mathbb{R}}^{n\times n}$ *. Then the secondary transpose (or secondary conjugate transpose* $A^{\theta }$ *in the case of complex matrices) of* A *denoted by* $A^{S}$ *and is defined as* $A^{S}=\left(b_{\mathrm{ij}}\right)$ *, where* $b_{\mathrm{ij}}=a_{n-j+1,n-i+1}$ *,* i,j=1,2,…n *.*

Definition 2
(Ref. 16) *Let* $A\in {\mathbb{C}}^{n\times n}$ *. Then the conjugate secondary transpose of* A *denoted by* $A^{\theta }$ *and is defined as* $A^{\theta }={\bar{A}}^{s}=\left(c_{\mathrm{ij}}\right)$ *where* $c_{\mathrm{ij}}={\bar{a}}_{n-j+1,n-i+1}$ *.*

The secondary transpose of a matrix is defined by reflecting the entries through sendary diagonal.

Definition 3
(Ref. 16) *A matrix is said to be secondary normal (S - normal) if* ${\mathrm{AA}}^{S}=A^{S}A$ *.*

Definition 4
(Ref. 12) $A^{{†}_{S}}$ *is said to be secondary generalized inverse of* A *if*

$${\mathrm{AA}}^{{†}_{S}}A=A\;A^{{†}_{S}}{\mathrm{AA}}^{{†}_{S}}=A^{{†}_{S}}\mathrm{and}{\mathrm{AA}}^{{†}_{S}}\mathrm{and}A^{{†}_{S}}A\mathrm{are}S-\mathrm{symmetric}.$$

Note that the matrix *A* is said to be secondary symmetric or S−symmetric if and only if $A^{S}=A$ .

Theorem 0.1
(Ref. 12) *Given an* m×n *matrix* A *. The following statements are equivalent.*

(1)
A *has S - cancellation property (i.e.,* $A^{s}\mathrm{AX}=0\mathrm{AX}=0$ *and* ${\mathrm{YAA}}^{s}=0\mathrm{YA}=0$ *).*
(2)
$\rho \left(A^{s}A\right)=\rho \left({\mathrm{AA}}^{s}\right)=\rho \left(A\right)$

(3)
$A^{{†}_{S}}$ *exists.*

Definition 5
(Ref. 17) *A matrix* $A\in {\mathbb{C}}^{n\times n}$ *is said to be EP (or range symmetric) if* $N\left(A\right)=N\left(A^{*}\right)$ *.*

Meenakshi ^3^ has defined EP in Minkowski space and has given equivalent conditions for a matrix to be range symmetric.

Let the components of a complex vector $C^{n}$ from 0 to *n*-1, be $u=\left(u_{0},u_{1},u_{2},\ldots ,u_{n-1}\right).$ Let us denote the Minkowski metric tensor by G and it is defined as $G=\left(u_{0},-u_{1},-u_{2},\ldots ,-u_{n-1}\right)$ . Now, the Minkowski metric matrix is $G=\left(\begin{array}{cc} 1 & 0\\ 0 & -I_{n-1} \end{array}\right)$ . The Minkowski inner product on $C^{n}$ is defined by $\left(u,v\right)=<u,\mathrm{Gv}>$ where <.,.> is the Hilbert inner product. A space with Minkowski inner product is defined as Minkowski space. The idea of Minkowski space arised when Xing ^18^ tried to study the optical devices described by the Mueller matrix which may not have a singluar value decomposition. The problem was solved by Renardy ^19^ by defining Minkowski space and obtained the singular value decomposition of Mueller matrix over the Minkowski space.

Definition 6
(Ref. 3) *A matrix* $A\in {\mathbb{C}}^{n\times n}$ *is said to be range symmetric in Minkowski space if and only if* $N\left(A\right)=N\left(A^{+}\right)$ *.*

Here $A^{+}$ represents the Minkowski adjoint given by $A^{+}={\mathrm{GA}}^{*}G$ where G is the Minkowski metric tensor.

## Results

In this section, we define secondary range symmetric matrices which is analogous to that of range symmetric matrices. Some equivalent conditions for a matrix to be range symmetric is also given here.

Definition 7
X *is secondary right (left) normalized g inverse of* A *where A* ∈ *R ^(n×n)^ if* AXA=A *,* XAX=X *and* AX *is* S *- symmetric. (* XA *is* S *- symmetric).*

Example 1:

Let $A=\left(\begin{array}{cc} 1 & 1\\ 2 & 1\\ 1 & 2 \end{array}\right).$ Note that the matrix $X=\left(\begin{array}{ccc} -1 & 1 & 0\\ 7/5 & -4/5 & 1/5 \end{array}\right)$ is both secondary right normalized g-inverse and secondary left normalized inverse of *A*. The conditions AXA=A,XAX=X can be easily verified.

Also $\mathrm{AX}=\left(\begin{array}{ccc} 2/5 & 1/5 & 1/5\\ -3/5 & 6/5 & 1/5\\ 9/5 & -3/5 & 2/5 \end{array}\right)={\left(\mathrm{AX}\right)}^{S}$ . Therefore *X* is secondary right normalized g-inverse of *A*.

Here $\mathrm{XA}=\left(\begin{array}{cc} 1 & 0\\ 0 & 1 \end{array}\right)={\left(\mathrm{XA}\right)}^{S}.$ Here *X* is also a left normalized g-inverse.

Definition 8
*Consider* $A\in {\mathbb{R}}^{m\times n}$ *. The S - transpose of* A *is defined as* $A^{s}=\left(b_{\mathrm{ij}}\right)$ *where* $b_{\mathrm{ij}}=a_{m-j+1,n-i+1}$ *,* 1≤i≤n,1≤j≤m *.*

Example 2:

Consider a matrix $A=\left(\begin{array}{ccc} 1 & 2 & 3\\ 4 & 5 & 6 \end{array}\right).$ The S – transpose of *A* is given by $A^{S}=\left(\begin{array}{cc} 6 & 3\\ 5 & 2\\ 4 & 1 \end{array}\right)$ .

Definition 9
*A matrix* $A\in {\mathbb{R}}^{n\times n}$ *secondary range symmetric if and only if* $N\left(A\right)=N\left(A^{S}\right)$

Example 3:

Let $A=\left(\begin{array}{cc} 1 & 0\\ 0 & 1 \end{array}\right).$ Here $N\left(A\right)\neq N\left(A^{*}\right).$ But $N\left(A\right)=N\left(A^{S}\right)$ . Therefore the matrix is not range symmetric, but it is secondary range symmetric.

Theorem 0.2
*Let* $A\in {\mathbb{R}}^{n\times n}$ *. Then the following conditions are equivalent.*

(1)
A *is secondary range symmetric*
(2)
VA *is EP*
(3)
AV *is EP* (V is a permutation matrix with ‘1’ in the secondary diagonal)
(4)
$N\left(A^{*}\right)=N\left(\mathrm{AV}\right)$

(5)
$C\left(A\right)=C\left(A^{S}\right)$

(6)
$A^{S}=\mathrm{BA}=\mathrm{AC}$ *where* B *and* C *are some nonsingular matrices*
(7)
$C\left(A^{*}\right)=C\left(\mathrm{VA}\right)$

(8)
$C\left(A^{*}\right)⊕N\left(A\right)={\mathbb{C}}^{n}$

(9)
$C\left(A\right)⊕N\left(A^{*}\right)={\mathbb{C}}^{n}$

Proof 1
$$\begin{array}{l} \text{A is secondary range symmetric}\Leftrightarrow N\left(A\right)=N\left(A^{S}\right)\\ \;\Leftrightarrow N\left(\mathrm{VA}\right)=N\left(A^{S}V\right)\;(\mathrm{since}\;V^{2}=I)\\ \;\Leftrightarrow \text{VA is EP}\\ \;\Leftrightarrow V\left(\mathrm{VA}\right)V^{S}\text{is EP}\\ \;\Leftrightarrow \text{AV is EP}. \end{array}$$

*Hence* $\left(1\right)\Leftrightarrow \left(2\right)\Leftrightarrow \left(3\right)$ *holds true.*

$\left(1\right)\Leftrightarrow \left(4\right)$

$$\begin{array}{l} \text{A is secondary range symmetric}\Leftrightarrow N\left(A\right)=N\left(A^{S}\right)\\ \;\Leftrightarrow N\left(A\right)=N\left({\mathrm{VA}}^{*}V\right)\;\text{(by definition of secondary transpose)}\\ \;\Leftrightarrow N\left(A\right)=N\left(A^{*}V\right)\\ \;\Leftrightarrow A^{*}V=A^{*}{\mathrm{VA}}^{\left(1\right)}A\\ \;\Leftrightarrow A^{*}=A^{*}{\mathrm{VA}}^{\left(1\right)}\mathrm{AV}\\ \;\Leftrightarrow A^{*}=A^{*}{\left(\mathrm{AV}\right)}^{\left(1\right)}\mathrm{AV}\\ \;\Leftrightarrow N\left(A^{*}\right)=N\left(\mathrm{AV}\right) \end{array}$$

*This proves the equivalence of* $\left(1\right)$ *and* $\left(4\right)$ *.*

$\left(3\right)\Leftrightarrow \left(5\right)$

$$\begin{array}{l} \text{AV is range symmetric}\Leftrightarrow C\left(\mathrm{AV}\right)=C{\left(\mathrm{AV}\right)}^{*}\\ \;\Leftrightarrow C\left(A\right)=C\left({\mathrm{VA}}^{*}\right)\;(\text{Since}\;V^{*}=V)\\ \;\Leftrightarrow C\left(A\right)=C\left({\mathrm{VA}}^{*}V\right)\\ \;\Leftrightarrow C\left(A\right)=C\left(A^{S}\right) \end{array}$$

*Hence* $\left(3\right)$ *and* $\left(5\right)$ *are equivalent.*

$\left(2\right)\Leftrightarrow \left(6\right)$

$$\begin{array}{l} \mathrm{VA}\;\text{is range symmetric}\Leftrightarrow {\left(\mathrm{VA}\right)}^{*}=\mathrm{VAP}\;\text{for some nonsingular}\;n\times n\;\text{matrix}\;P\\ \;\Leftrightarrow A^{*}V=\mathrm{VAP}\\ \;\Leftrightarrow {\mathrm{VA}}^{*}V=\mathrm{AP}\\ \;\Leftrightarrow A^{S}=\mathrm{AP}\\ \;\Leftrightarrow A={\left(\mathrm{AP}\right)}^{S}=P^{S}A^{S}\;\text{(By property of secondary transpose)}\\ \;\Leftrightarrow A^{S}={\left(P^{S}\right)}^{-1}A\\ \;\Leftrightarrow A^{S}=\mathrm{KA}\;\text{where}\;K={\left(P^{S}\right)}^{-1}.\;(\text{Using}{\left(A^{{†}_{S}}\right)}^{{†}_{S}}=A) \end{array}$$

*Hence* $\left(2\right)\Leftrightarrow \left(6\right)$ .

$\left(5\right)\Leftrightarrow \left(7\right)$

$$\begin{array}{l} C\left(A\right)=C\left(A^{S}\right)\Leftrightarrow C\left(A\right)=C\left({\mathrm{VA}}^{S}V\right)\\ \;\Leftrightarrow C\left(A\right)=C\left({\mathrm{VA}}^{S}\right)\\ \;\Leftrightarrow {\mathrm{VA}}^{*}={\mathrm{AA}}^{\left(1\right)}{\mathrm{VA}}^{*}\;(\mathrm{By}[14])\\ \;\Leftrightarrow A^{*}={\mathrm{VAA}}^{\left(1\right)}{\mathrm{VA}}^{*}\\ \;\Leftrightarrow A^{*}=\left(\mathrm{VA}\right){\left(\mathrm{VA}\right)}^{\left(1\right)}A^{*}\\ \;\Leftrightarrow C\left(A^{*}\right)=C\left(\mathrm{VA}\right) \end{array}$$

*Thus equivalence of* $\left(5\right)$ *and* $\left(7\right)$ *are proved.*

$\left(2\right)$ ⇔ $\left(8\right)$ .

$$\begin{array}{l} \text{VA is range symmetric}\Leftrightarrow {\mathbb{C}}^{n}=C\left(\mathrm{VA}\right)⊕N\left(\mathrm{GA}\right)\\ \;=C\left({\left(\mathrm{VA}\right)}^{*}\right)⊕N\left(A\right)\\ \;=C\left(A^{*}V\right)⊕N\left(A\right)\\ \;=C\left(A^{*}\right)⊕N\left(A\right) \end{array}$$

*Thus equivalence of* $\left(2\right)$ *and* $\left(8\right)$ *are proved.*

$\left(3\right)\Leftrightarrow \left(9\right)$

$$\begin{array}{l} \text{AV is range symmetric}\Leftrightarrow {\mathbb{C}}^{n}=C\left(\mathrm{AV}\right)⊕N\left(\mathrm{AV}\right)\\ \;={\mathbb{C}}^{n}=C\left(\mathrm{AV}\right)⊕N\left({\left(\mathrm{AV}\right)}^{*}\right)\\ \;=C\left(A\right)⊕N\left(A^{*}\right) \end{array}$$

*Thus the equivalence of* $\left(3\right)$ *and* $\left(9\right)$ *is proved.*

From the following example, it is clear that EP matrices in Minkowski space defined by Meenakshi ^3^ and secondary range symmetric matrices are two different concepts.

Consider a matrix $A=\left(\begin{array}{cc} -1 & 1\\ 1 & -1 \end{array}\right)$ where $A^{S}=\left(\begin{array}{cc} -1 & 1\\ 1 & -1 \end{array}\right)$ . Note that, here the secondary transpose *A* ^*S*^ of the matrix *A* coincides with *A*. Clearly *A* is secondary range symmetric (i.e., $N\left(A\right)=N\left(A^{S}\right).$ Here, A is secondary normal as well as $\rho \left(A\right)=\rho \left({\mathrm{AA}}^{S}\right)$ . However, the matrix is not range symmetric in Minkowski’s space since $A^{+}={\mathrm{GA}}^{*}G=\left(\begin{array}{cc} -1 & -1\\ -1 & -1 \end{array}\right)$ . It is clear that $N\left(A\right)\neq N\left(A^{+}\right)$ .

In this example, the matrix A is secondary range symmetric, but not range symmetric in Minkowski space.

In the following example, B is range symmetric in Minkowski space. But it is not secondary range symmetric.

Let $B=\left(\begin{array}{cc} 1 & -1\\ 1 & -1 \end{array}\right)$ and $B^{S}=\left(\begin{array}{cc} -1 & -1\\ 1 & 1 \end{array}\right)$ . Clearly B is not secondary range symmetric.

Observe that B is range symmetric in Minkowski space. Since, $B^{+}={\mathrm{GB}}^{*}G=\left(\begin{array}{cc} 1 & -1\\ 1 & -1 \end{array}\right)$ so that $N\left(B\right)=N\left(B^{+}\right)$ .

These examples shows that secondary range symmetric matrices and range symmetric matrices in Minkowski inverse are two different matrices even though the proof techniques adopted here are similar.

Note that $A^{S}={\mathrm{VA}}^{*}V={\mathrm{VGA}}^{+}\mathrm{GV}$ .

A necessary condition for a matrix to be a S - EP (secondary range symmetric) is proved here.

Theorem 0.3
*Let* $A\in {\mathbb{R}}^{n\times n}$ *. If* A *is secondary normal and* $\rho \left(A\right)=\rho \left({\mathrm{AA}}^{S}\right)$ *, then* A *is secondary range symmetric.*

Proof 2
*Since* A *is secondary normal,* ${\mathrm{AA}}^{S}=A^{S}A$ *. Hence* $\rho \left(A\right)=\rho \left({\mathrm{AA}}^{S}\right)=\rho \left(A^{S}A\right)=\rho \left(A^{S}\right)$ *which implies* $N\left(A\right)=N\left({\mathrm{AA}}^{S}\right)=N\left(A^{S}A\right)=N\left(A^{S}\right)$ *. Thus* A *is secondary range symmetric.*

A relation connecting range symmetric and secondary range symmetric matrices is given below:

Theorem 0.4
*Let* $A\in {\mathbb{R}}^{n\times n}$ *. Then any two of the following conditions imply the third one.*

(1)
A *is EP.*
(2)
A *is secondary EP.*
(3)
$C\left(A\right)=C\left(\mathrm{VA}\right)$ *.*

Proof 3
$\left(1\right),\left(2\right)\Rightarrow \left(3\right)$

*Since* A *is EP,* $C\left(A\right)=C\left(A^{*}\right)$ *. By condition (7) of* *Theorem 0.2* *,* $C\left(A^{*}\right)=C\left(\mathrm{VA}\right)$ *as* A *is secondary range symmetric. Hence* A *is secondary EP* ⇔ $C\left(A\right)=C\left(\mathrm{VA}\right)$ *.*

(1), (3) ⇒ (2)

*Since A is EP,* $C\left(A\right)=C\left(A^{*}\right).$ *Also, from (3) we have* $C\left(A\right)=C\left(\mathrm{VA}\right),$ *which gives* $C\left(A^{*}\right)=C\left(\mathrm{VA}\right).$ *Hence, by* *Theorem 0.2* *, A is secondary range symmetric.*

$\left(2\right),\left(3\right)\Rightarrow \left(1\right)$

*Since* A *is S - EP, by condition (2) of* *Theorem 0.2* *,* VA *is range symmetric. Hence* $C\left(\mathrm{VA}\right)=C\left({\left(\mathrm{VA}\right)}^{*}\right)=C\left(A^{*}V\right)=C\left(A^{*}\right)$ *. Also, By (3),* $C\left(A\right)=C\left(\mathrm{VA}\right)$ *from which it follows that* $C\left(A\right)=C\left(A^{*}\right)$ *. Hence* A *is range symmetric. Thus (1) holds.*

For any square complex matrix A , there exists unique S - symmetric matrices such that A=M+iN where $M=\frac{1}{2}\left(A+A^{S}\right)$ and $N=\frac{1}{2i}\left(A-A^{S}\right)$ . In the following theorem, an equivalent condition for a matrix A to be secondary range symmetric is obtained interms of M , the S -symmetric part of A .

Theorem 0.5
*For* $A\in {\mathbb{R}}^{n\times n}$ *,* A *is secondary range symmetric if and only if* $N\left(A\right)\subseteq N\left(M\right)$ *where* M *is the S - symmetric part of* A *.*

Proof 4
*If* A *is secondary range symmetric, then* $N\left(A\right)=N\left(A^{S}\right)$ *. For* $x\in N\left(A\right)$ *,* Ax=0 *and* $A^{S}x=0$ *. Hence* Mx=0 *. Thus,* $N\left(A\right)\subseteq N\left(M\right)$ *, then* Ax=0⇒Mx=0 *and hence* Nx=0 *. Therefore* $N\left(A\right)\subseteq N\left(N\right)$ *. Thus* $N\left(A\right)\subseteq N\left(M\right)\cap N\left(N\right)$ *. Since, both* M *and* N *are* S *-symmetric, they are secondary range symmetric.*

$$N\left(M\right)=N\left(M^{S}\right)=N\left({\mathrm{VM}}^{*}V\right)=N\left(M^{*}V\right)$$

*and*

$$N\left(N\right)=N\left(N^{S}\right)=N\left({\mathrm{VN}}^{*}V\right)=N\left(N^{*}V\right)$$

*Now*, $N\left(A\right)\subseteq N\left(M\right)\cap N\left(N\right)=N\left(M^{*}V\right)\cap N\left(N^{*}V\right)\subseteq N\left[\left(M^{*}+{\text{iN}}^{*}\right)V\right]=N\left(A^{*}V\right)$ and $\rho \left(A\right)=\rho \left(A^{*}\right)=\rho \left(A^{*}V\right)$ . *Therefore*, $N\left(A\right)=N\left(A^{*}V\right)=N\left({\mathrm{VA}}^{*}V\right)=N\left(A^{S}\right)$ . *Thus* A *is secondary range symmetric.*

We shall discuss the existence of secondary generalized inverse inverse of a secondary range symmetric matrix. First, we shall prove certain lemmas, to simplify the proof of the main result.

Lemma 1
*For an* m×n *matrix* A *, if* $A^{{†}_{S}}$ *exists, then* $C\left(A^{{†}_{S}}\right)=C\left(A^{S}\right)$ *.*

Proof 5
*If* $A^{{†}_{S}}$ *exists, then* $A^{{†}_{S}}A={\left(A^{{†}_{S}}A\right)}^{S}=A^{S}{\left({\mathrm{AA}}^{{†}_{S}}\right)}^{S}{\left(A^{{†}_{S}}\right)}^{S}$ *and*

$$A^{{†}_{S}}=A^{{†}_{S}}{\mathrm{AA}}^{{†}_{S}}=A^{S}{\left(A^{{†}_{S}}\right)}^{S}A^{{†}_{S}}\Rightarrow C\left(A^{{†}_{S}}\right)\subseteq C\left(A^{S}\right).$$

*Further*, $\rho \left(A^{{†}_{S}}\right)=\rho \left(A\right)=\rho \left(A^{S}\right)$ . *Thus*, $C\left(A^{{†}_{S}}\right)=C\left(A^{S}\right)$ .

Lemma 2
*For an* m×n *matrix* A *, if* $A^{{†}_{S}}$ *exists, then* $C\left({\mathrm{AA}}^{{†}_{S}}\right)$ *is the projection on* $C\left(A^{{†}_{S}}\right)$ *and* $A^{{†}_{S}}A$ *is the projection on* $C\left(A^{{†}_{S}}\right)$ *.*

Proof 6
$x\in C\left(A\right)$ *if and only if* $x=\mathrm{Ay}={\mathrm{AA}}^{{†}_{S}}\mathrm{Ay}={\mathrm{AA}}^{{†}_{S}}x$ *. By* *definition 2* *,* ${\mathrm{AA}}^{{†}_{S}}$ *being S - symmetric, idempotent is the projection on* $C\left(A\right)$ *. Similarly,* $x\in C\left(A^{{†}_{S}}\right)$ *if and only if* $x=A^{{†}_{S}}{\mathrm{AA}}^{{†}_{S}}y=A^{{†}_{S}}\mathrm{Ax}$ *and* $A^{{†}_{S}}A$ *is S - symmetric and idempotent. Hence* $A^{{†}_{S}}A$ *is the projection on* $C\left(A^{{†}_{S}}\right)$ *.*

Theorem 0.6
*For an* n×n *matrix* A *, the following are equivalent:*

(1)
A *is secondary range symmetric and* $\rho \left(A\right)=\rho \left(A^{2}\right).$

(2)
$A^{{†}_{S}}$ *exists and* $A^{{†}_{S}}$ *is secondary range symmetric.*
(3) *There exists a symmetric idempotent matrix E such that* AE=EA *and* $C\left(A\right)=C\left(E\right).$

Proof 7
$\left(1\right)\Rightarrow \left(2\right)$ *. Since* $\rho \left(A\right)=\rho \left(A^{2}\right)$ *and* A *is secondary symmetric, by using* *Theorem 0.2* *we have,* $\rho \left(A^{S}A\right)=\rho \left({\mathrm{BA}}^{2}\right)=\rho \left(A^{2}\right)=\rho \left(A\right)$ *and* $\rho \left({\mathrm{AA}}^{S}\right)=\rho \left(A^{2}C\right)=\rho \left(A^{2}\right)=\rho \left(A\right)$ *. Thus* $\rho \left(A\right)=\rho \left({\mathrm{AA}}^{S}\right)=\rho \left(A^{S}A\right)$ *. Hence by* *Thoerem 0.1* *, it follows that* $A^{{†}_{S}}$ *exists, By* *Lemma 1* *and* *Theorem 0.2* *,* $C\left(A^{{†}_{S}}\right)=C\left(A^{S}\right)=C\left(A\right)=C\left({\left(A^{S}\right)}^{S}\right)=C\left({\left(A^{{†}_{S}}\right)}^{S}\right)$ *. Hence* $A^{{†}_{S}}$ *is range symmetric. Thus (2) holds.*

$\left(2\right)\Rightarrow \left(3\right)$ . *Since* $A^{{†}_{S}}$ *exists, by* Lemma 1, $C\left(A^{{†}_{S}}\right)=C\left(A^{S}\right)$ , by equivalence of condition (1) and (5) of Theorem 0.2, $A^{{†}_{S}}$ *is secondary range symmetric which implies that* $C\left(A^{{†}_{S}}\right)=C\left({\left(A^{{†}_{S}}\right)}^{S}\right)$ . *Hence* $C\left(A^{S}\right)=C\left({\left(A^{{†}_{S}}\right)}^{S}\right)$ . By Lemma 2, *it follows that* $A^{S}{\left(A^{S}\right)}^{{†}_{S}}={\left(A^{{†}_{S}}\right)}^{S}A^{S}$ , *hence* ${\left(A^{{†}_{S}}A\right)}^{S}={\left({\mathrm{AA}}^{{†}_{S}}\right)}^{S}$ . *By* definition 2, $A^{{†}_{S}}A={\mathrm{AA}}^{{†}_{S}}=E$ , *is* S - *symmetric, idempotent and* AE=EA=A ; *hence* $C\left(A\right)\subseteq C\left(E\right)$ and $\rho \left(E\right)=\rho \left({\mathrm{AA}}^{{†}_{S}}\right)=\rho \left(A\right)$ , *which implies* $C\left(A\right)=C\left(E\right)$ . *Thus* $\left(3\right)$ *holds.*

$\left(3\right)\Rightarrow \left(1\right)$ . *Since*, E *is S - symmetric and idempotent*, $E^{S}=E=E^{2}$ , *by* lemma 1.1, $E^{{†}_{S}}$ *exists and* $E^{{†}_{S}}=E$ *implies* E *is the projection on* $C\left(A\right)$ . *For all reflexive g-inverses* $A^{r}$ of A , ${\mathrm{AA}}^{r}={\mathrm{EE}}^{{†}_{S}}=E$ . *Since* E *is S - symmetric and idempotent*, ${\mathrm{AA}}^{r}$ *is* S - *symmetric. Hence by* definition 7, $A^{n}$ *exists and* ${\mathrm{AA}}^{n}={\mathrm{EE}}^{{†}_{S}}=E$ *which implies* EA=A . *By hypothesis* AE=EA=A . *Therefore* ${\mathrm{AA}}^{n}=A^{n}A=E$ . *Thus both* ${\mathrm{AA}}^{n}$ *and* $A^{n}A$ *are S - symmetric*. *By* definition 2, $A^{{†}_{S}}$ *exists and* $E={\mathrm{AA}}^{{†}_{S}}=A^{{†}_{S}}A$ . *By taking secondary transpose on* AE=EA=A , *we* get ${\mathrm{EA}}^{S}=A^{S}E=A^{S}$ . $C\left(A^{S}\right)\subseteq C\left(E\right)=C\left(A\right)$ *and* $\rho \left(A^{S}\right)=\rho \left(A\right)$ . *Therefore* $C\left(A\right)=C\left(A^{S}\right)$ . *By* theorem 0.2, A *is secondary ramge symmetric.* $\rho \left({\mathrm{AA}}^{S}\right)\geq \rho \left({\mathrm{AA}}^{S}{\left(A^{{†}_{S}}\right)}^{S}\right)=\rho \left(A{\left(A^{{†}_{S}}\right)}^{S}\right)$ , $\rho \left(\mathrm{AE}\right)=\rho \left(A\right)\geq \rho \left({\mathrm{AA}}^{S}\right)$ . *Thus* $\rho \left(A\right)=\rho \left({\mathrm{AA}}^{S}\right)=\rho \left(A^{2}C\right)=\rho \left(A^{2}\right)$ . *Thus* (1) *holds.* *Hence the theorem.*

Corollary 1
*Let A be* n×n *secondary range symmetric matrix. Then exists* $A^{{†}_{S}}$ *if and only if* $\rho \left(A\right)=\rho \left(A^{2}\right)$ *.*

Proof 8
*Since* A *is secondary range symmetric and* $\rho \left(A\right)=\rho \left(A^{2}\right)$ *, the existence of* $A^{{†}_{S}}$ *follows from equivalence of (1) and (2) of Thereom 0.6. Conversly, if* A *is secondary range symmetric, and* $A^{{†}_{S}}$ *exists, then by equivalence of (2) and (3) of Theorem 0.1* $\rho \left(A\right)=\rho \left({\mathrm{AA}}^{S}\right)=\rho \left(A^{S}A\right)$ *and by* *Theorem 0.2* *,* $A^{S}=\mathrm{AC}$ *. Hence* $\rho \left(A\right)=\rho \left(\mathrm{AAC}\right)=\rho \left(A^{2}\right)$ *.*

## Conclusion

In this article we defined and characterized the concept of secondary range symmetric matrices. The Moore Penrose inverse exists for any matrix. But, in the case of secondary generalized inverse this is not true. Here, we obtained a necessary condition for a secondary range symmetric matrix to have an s-g inverse. In fact this condition holds true for the existence of secondary generalized inverse for any matrix.

As an extension of this work, the sum of range symmetric matrices are discussed in Ref. 20. One can think of defining weighted secondary EP matrices and its characterizations. Also, extending secondary range symmetric matrix to indefinite inner product spaces will open up a new area of research.
