# Supplementary material for: Secondary range symmetric matrices
Source: F1000Res. 2024 Sep 11;13:112. Originally published 2024 Feb 19. [Version 2] doi: 10.12688/f1000research.144171.2 (PMC11162518; doi:10.12688/f1000research.144171.2)
Supplement: Peer review history

# Reviewer report: Reviewer response for version 2

**DOI:** 10.5256/f1000research.168149.r322688

**Published:** 2024-10-11

**Authors:** Pankaj Kumar Manjhi

**Affiliations:** 1 Department of Mathematics,, Vinoba Bhave University,, Hazaribag,, Jharkhand,, India

The author has successfully addressed all the provided suggestions, resulting in a significantly improved manuscript. The revised version is now clearer, more comprehensible, and effectively presents the research. It is suitable for proceeding to the next stage of the publication process.

Is the work clearly and accurately presented and does it cite the current literature?

Yes

If applicable, is the statistical analysis and its interpretation appropriate?

Yes

Are all the source data underlying the results available to ensure full reproducibility?

Partly

Is the study design appropriate and is the work technically sound?

Partly

Are the conclusions drawn adequately supported by the results?

Partly

Are sufficient details of methods and analysis provided to allow replication by others?

Partly

Reviewer Expertise:

NA

I confirm that I have read this submission and believe that I have an appropriate level of expertise to confirm that it is of an acceptable scientific standard.

# Reviewer report: Reviewer response for version 1

**DOI:** 10.5256/f1000research.157925.r261048

**Published:** 2024-06-08

**Authors:** Mehsin Jabel Atteya

**Affiliations:** 1 Department of Mathematics, Al- Mustansiriyah University, Falastin St, Baghdad, Iraq

Dear Sir.,

 My Report:

 1. The Definitions 7, 8 and 9 need to provide examples.

 2. In the proof of Theorem 0.4, where the prove of the case (1) and (3) implies to (2).

 3. in the proof of Corollary 1, the author used Theorem 0.1 while Theorem 0.1 concerning *an* m×n *matrix* A.

 4. Again in Corollary 0.1, the author depended on Theorems (0.1 and 0.6) without mention the branches.

 Similarly for the prove of Theorems(0.3 and 0.6) where the author used Theorems (0.2 and 0.1).

 5. The branches of Theorem (0.2) wrote by Roman numbers system while in the proof used *Arabic* numbers system.

Is the work clearly and accurately presented and does it cite the current literature?

Yes

If applicable, is the statistical analysis and its interpretation appropriate?

Not applicable

Are all the source data underlying the results available to ensure full reproducibility?

Yes

Is the study design appropriate and is the work technically sound?

Yes

Are the conclusions drawn adequately supported by the results?

Yes

Are sufficient details of methods and analysis provided to allow replication by others?

Yes

Reviewer Expertise:

The paper tried to supply new results with avoid any mistake.

I confirm that I have read this submission and believe that I have an appropriate level of expertise to confirm that it is of an acceptable scientific standard.

# Response

**Published:** 2024-06-19

**Authors:** Divya Shenoy

**Affiliations:** Mathematics, Manipal Academy of Higher Education, Manipal, Karnataka, India

**Comments and Responses:**

**Comment 1:** The Definitions 7, 8 and 9 need to provide examples.

**Response:  **Definitions 7,8 and 9 are illustrated with examples- 1, 2 and 3 respectively.

**Comment 2:** In the proof of Theorem 0.4, where the prove of the case (1) and (3) implies to (2).

**Response: **The omission error has been corrected in the revised article. A proof for (1) and (3) implies (2) is added in the new version.

**Comment 3:** In the proof of Corollary 1, the author used Theorem 0.1 while Theorem 0.1 concerning *an* m×n *matrix* A.

**Response: **Theorem 0.1 holds for a matrix of any order [m×n  as well as n×n ].

 For generalization, the authors [in reference: V. Savitha, D. P. Shenoy, K. Umashankar and R. B. Bapat, Secondary transpose of a matrix and generalized inverses, Journal of Algebra and its applications, 23(3), 2450052, 2024, doi:10.1142/S021949882450052X.] have considered rectangular matrices. Similar to *Moore-Penrose inverse*, in this context, the conditions for rectangular matrices holds true for square matrices also.

**Comment 4:** Again in Corollary 0.1, the author depended on Theorems (0.1 and 0.6) without mention the branches. Similarly for the prove of Theorems(0.3 and 0.6) where the author used Theorems (0.2 and 0.1).

**Response: **The appropriate branches are mentioned in the revised version. 

**Comment 5:** The branches of Theorem (0.2) wrote by Roman numbers system while in the proof used *Arabic* numbers system.

**Response: **The typological errors have been corrected.

# Reviewer report: Reviewer response for version 1

**DOI:** 10.5256/f1000research.157925.r274406

**Published:** 2024-06-08

**Authors:** Pankaj Kumar Manjhi

**Affiliations:** 1 Department of Mathematics,, Vinoba Bhave University,, Hazaribag,, Jharkhand,, India

I am very delighted to see the scholarly interest in the study of range symmetric matrices. Here are my suggestions for improving the article:

1. Some definitions (such as definition 1) are not clearly written and need to be clarified for better understanding.
2. The article requires a sufficient number of illustrations to explain the theory and definitions, which are currently missing.
3. A new preliminary section should be included, containing all the necessary basic concepts to provide a strong foundation for the reader.
4. Definition 7 does not adequately explain the entries of matrix A and needs to be revised for clarity.
5. Definitions 7 and 8 should be illustrated through examples to enhance comprehension.
6. The proofs, especially the proof of Theorem 1, are not written in an easily understandable manner and should be simplified for better readability.
7. A conclusion and discussion section must be included and well explained to summarize the findings and implications of the study.
8. The definition of Minkowski’s space should be included, accompanied by examples to illustrate its application.
9. The examples given at the beginning of page 5 are not properly explained and need to be elaborated for clarity.

By addressing these points, the article can be significantly improved and provide a clearer, more comprehensive understanding of range symmetric matrices.

Is the work clearly and accurately presented and does it cite the current literature?

Yes

If applicable, is the statistical analysis and its interpretation appropriate?

Yes

Are all the source data underlying the results available to ensure full reproducibility?

Partly

Is the study design appropriate and is the work technically sound?

Partly

Are the conclusions drawn adequately supported by the results?

Partly

Are sufficient details of methods and analysis provided to allow replication by others?

Partly

Reviewer Expertise:

Combinatorial matrices, Discrete Mathematics, computer science

I confirm that I have read this submission and believe that I have an appropriate level of expertise to confirm that it is of an acceptable scientific standard, however I have significant reservations, as outlined above.

# Response

**Published:** 2024-06-19

**Authors:** Divya Shenoy

**Affiliations:** Mathematics, Manipal Academy of Higher Education, Manipal, Karnataka, India

**Comments and Responses:**

**Comment 1: **Some definitions (such as definition 1) are not clearly written and need to be clarified for better understanding.

**Response: **Definition 1 (of the secondary transpose of a rectangular matrix) has been quoted from: A. Lee, Secondary symmetric, Skew symmetric and orthogonal matrices, Period. Math. Hungar 7(1), pp. 63-70, 1976.

 In the revised article, it is elaborated and an example [example-2, *results*] has been added for better understanding of the concept.

**Comment 2:** The article requires a sufficient number of illustrations to explain the theory and definitions, which are currently missing.

**Response: **As per the suggestion, the article is modified providing sufficient examples. On page 3, the definitions are explained with examples [example 1, 2 and 3]. Also, a clearer explanation is given for the example given after the proof of theorem 0.1.

**Comment 3:** A new preliminary section should be included, containing all the necessary basic concepts to provide a strong foundation for the reader.

**Response: **A *preliminary section* [ *section 2*] is included with all necessary basic concepts.

**Comment 4:** Definition 7 does not adequately explain the entries of matrix A and needs to be revised for clarity.

**Response: **The definition (7) has been revised. The entries of the matrix are from a real field. The explanation has been provided in the text.

**Comment 5:** Definitions 7 and 8 should be illustrated through examples to enhance comprehension.

**Response:** As per the suggestion, definitions 7 and 8 are explained with examples.

**Comment 6:** The proofs, especially the proof of Theorem 1, are not written in an easily understandable manner and should be simplified for better readability.

**Response: **Explanation for the steps for the proof of Theorem -1, has been provided within parenthesis.

**Comment 7:** A conclusion and discussion section must be included and well explained to summarize the findings and implications of the study.

**Response:  **The *conclusion* section has been modified to include discussion, and scope for future work.

**Comment 8:** The definition of Minkowski’s space should be included, accompanied by examples to illustrate its application

**Response: **As per the suggestion, the definition of Minkowski space, with application and reference is included in the *preliminary section*.

**Comment 9: **The examples given at the beginning of page 5 are not properly explained and need to be elaborated for clarity.

**Response:** The example is elaborated for better understanding.

# Reviewer report: Reviewer response for version 1

**DOI:** 10.5256/f1000research.157925.r252908

**Published:** 2024-03-28

**Authors:** P Sam Johnson

**Affiliations:** 1 National Institute of Technology Karnataka, Surathkal, Surathkal, Karnataka,, India

The paper is well written.  It discusses secondary range symmetric matrices.  It should incorporate the following suggestions / corrections :

**Minor points :**

 (i) First line of abstract should be "is" introduced.

 (ii) No uniformity of symbol "s" in many places : s-symmetric, s-transpose ; capital letter or a small letter

 (iii) Corollary 1 in page 6 should be rephrased as : Let A be an nxn secondary range symmetric matrix.

**Major point:**

 (iv) The following recent references may be added.

Is the work clearly and accurately presented and does it cite the current literature?

No

If applicable, is the statistical analysis and its interpretation appropriate?

Not applicable

Are all the source data underlying the results available to ensure full reproducibility?

Yes

Is the study design appropriate and is the work technically sound?

Yes

Are the conclusions drawn adequately supported by the results?

Yes

Are sufficient details of methods and analysis provided to allow replication by others?

Yes

Reviewer Expertise:

Functional Analysis

I confirm that I have read this submission and believe that I have an appropriate level of expertise to confirm that it is of an acceptable scientific standard.

## References

[1] : Product and factorization of hypo-EP operators. *Special Matrices* .2018;6(1) : 10.1515/spma-2018-0030 376-382 10.1515/spma-2018-0030

[2] : Closed EP and hypo-EP operators on Hilbert spaces. *The Journal of Analysis* .2022;30(4) : 10.1007/s41478-022-00401-5 1377-1390 10.1007/s41478-022-00401-5

# Response

**Published:** 2024-03-31

**Authors:** Divya Shenoy

**Affiliations:** Mathematics, Manipal Academy of Higher Education, Manipal, Karnataka, India

I thank reviewer 1 for the critical comments.

 The suggested corrections (typo errors) and the inclusion of latest references in the manuscript and their corresponding information in the body of the manuscript will be incorporated in version 1, which will be uploaded after receiving comments from the second reviewer.

# Response

**Published:** 2024-06-19

**Authors:** Divya Shenoy

**Affiliations:** Mathematics, Manipal Academy of Higher Education, Manipal, Karnataka, India

**Comments and Responses**

**A. Response to Minor points :**

**Comment (i): **First line of abstract should be "is" introduced.

**Response**: The correction has been incorporated.

**Comment (ii):** No uniformity of symbol "s" in many places: s-symmetric, s-transpose; capital letter or a small letter.

**Response: **Uniformity has been maintained in the new version. Capital letter is used for all instances, S – symmetric, S – transpose etc.

**Comment (iii):** Corollary 1 in page 6 should be rephrased as: Let A be an nxn secondary range symmetric matrix.

**Response: **The suggested correction has been incorporated.

**Major point:**

**Comment (iv):** Regarding the addition of recent references on a) product and factorization of hypo-EP operators and b) Closed EP and hypo-EP operators on Hilbert spaces. 

**Response: **The latest references have been added. The relevant text is added in the article, with appropriate citation.
